# Bacterial larvicides used for malaria vector control in sub-Saharan Africa: review of their effectiveness and operational feasibility

**DOI:** 10.1186/s13071-019-3683-5

**Published:** 2019-08-30

**Authors:** Yahya A. Derua, Eliningaya J. Kweka, William N. Kisinza, Andrew K. Githeko, Franklin W. Mosha

**Affiliations:** 1 Kilimanjaro Christian Medical University College, Tumaini University Makumira, Moshi, Tanzania; 2 National Institute for Medical Research, Amani Research Centre, Muheza, Tanga Tanzania; 3 Division of Livestock and Human Diseases Vector Control, Tropical Pesticides Research Institute, Arusha, Tanzania; 4 Department of Medical Parasitology and Entomology, Catholic University of Health and Allied Sciences, Mwanza, Tanzania; 5 Climate and Human Health Research Unit, Centre for Global Health Research, Kenya Medical Research Institute, Kisumu, Kenya

**Keywords:** Bacterial larvicides, *Bacillus thuringiensis* var. *israelensis*, *Bacillus sphaericus*, *Anopheles gambiae* (*sensu lato*), *Anopheles gambiae* (*sensu stricto*), *Anopheles arabiensis*, *Anopheles funestus*, Sub-Saharan Africa

## Abstract

Several trials and reviews have outlined the potential role of larviciding for malaria control in sub-Saharan Africa (SSA) to supplement the core indoor insecticide-based interventions. It has been argued that widespread use of long-lasting insecticide-treated nets (LLINs) and indoor residual spraying (IRS) interventions in many parts of Africa result in many new areas with low and focal malaria transmission that can be targeted with larvicides. As some countries in SSA are making good progress in malaria control, larval source management, particularly with bacterial larvicides, could be included in the list of viable options to maintain the gains achieved while paving the way to malaria elimination. We conducted a review of published literature that investigated the application of bacterial larvicides, *Bacillus thuringiensis* var. *israelensis* (*Bti*) and/or *Bacillus sphaericus* (*Bs*) for malaria vector control in SSA. Data for the review were identified through PubMed, the extensive files of the authors and reference lists of relevant articles retrieved. A total of 56 relevant studies were identified and included in the review. The findings indicated that, at low application rates, bacterial larvicide products based on *Bti* and/or *Bs* were effective in controlling malaria vectors. The larvicide interventions were found to be feasible, accepted by the general community, safe to the non-target organisms and the costs compared fairly well with those of other vector control measures practiced in SSA. Our review suggests that larviciding should gain more ground as a tool for integrated malaria vector control due to the decline in malaria which creates more appropriate conditions for the intervention and to the recognition of limitations of insecticide-based vector control tools. The advancement of new technology for mapping landscapes and environments could moreover facilitate identification and targeting of the numerous larval habitats preferred by the African malaria vectors. To build sustainable anti-larval measures in SSA, there is a great need to build capacity in relevant specialties and develop organizational structures for governance and management of larval source management programmes.

## Background

Malaria mosquito vector control in sub-Saharan Africa (SSA) relies on the use of insecticide-treated bednets and/or indoor residual spraying with insecticide. These interventions have been shown to be effective and the recent decline in malaria prevalence in many parts of Africa has been attributed in part to their wide-scale use for mosquito control [1]. However, emerging and widespread insecticide resistance threatens the success made with these tools [2–4]. In addition, insecticide-based interventions have been reported to be the major drive towards the observed behavioral adaptation by malaria vectors [5, 6]. To maintain the gains achieved in malaria control over the last decade, it is crucial to implement measures that will mitigate insecticide resistance and behavioral adaptation by malaria vectors [7].

Mosquito larval control interventions have proven records of lowering malaria transmission and even eradication of malaria mosquitoes [8]. It has been shown that unlike adult mosquitoes, larvae cannot change their behavior to avoid a control intervention targeted at larval habitats [9]. Moreover, a larval control strategy also serves to extend the useful life of insecticides against adult mosquitoes by reducing the size of the population being selected for resistance and the strategy is equally effective in controlling both indoor and outdoor biting mosquitoes. Integrating larval source reduction with adult mosquito control interventions like insecticide-treated bednets has therefore been considered to be a highly effective strategy to control malaria [10]. Larviciding with chemical agents was historically an important component of malaria vector control [8, 11]. However, due to significant adverse effects to other non-target species, chemical larvicides have received less attention in the past decades. Instead, preference has been shifted to the use of microbial larvicides *Bacillus thuringiensis* var. *israelensis* (*Bti*) and *Bacillus sphaericus* (*Bs*), which selectively kill mosquito larvae with negligible effect to non-target organisms [12].

Despite the proven role of larval control and the historical success of such interventions in malaria control, larviciding has remained largely neglected for malaria control in SSA [13]. The World Health Organization (WHO) recommends larviciding to be used in moderate to low transmission settings as a supplement to core interventions (long-lasting insecticide-treated nets [LLINs] and indoor residual spraying [IRS]) in settings where larval habitats are few, fixed and findable, such as urban areas, desert fringes, high altitudes and rural areas with high population densities [14]. Of particular relevance to this recommendation, it has been argued that intensification of LLINs and IRS interventions in many parts of Africa will result in many new areas with low and focal malaria transmission [14]. Moreover, the WHO calls for a search for viable supplementary strategies for managing vector-borne diseases and reducing reliance on chemical insecticides [15]. Likewise, it has been argued that the current malaria control interventions constitute a necessary but insufficient set of measures to ensure a sustainable control and thus larviciding could play an important role when other vector control interventions have achieved their maximum practical impact [14].

In the past decades, there has been growing evidence suggesting that larval source management by applying bacterial larvicides *Bti* and *Bs* has the potential to lower the density of mosquito vectors, as previously summarized [12, 16]. The efficacy and safety of *Bti* and *Bs* have been reported to be high making them ideal for inclusion in the integrated vector management (IVM) programmes for mosquito-borne disease control [12]. However, control efficacy of *Bti* and *Bs* has also been reported to vary greatly, mainly due to factors related to target mosquitoes (species of mosquito, their respective feeding strategies, age and density of larvae), larval habitat conditions (temperature, solar radiation, depth of water, turbidity, organic contents and presence of vegetation) and larvicide properties (application rates, toxin contents, type of carrier, how effective the material reach the target, settling rate, means of application and frequent of treatment) [17]. Due to this heterogeneity of their activity, the general consensus suggests that a larviciding strategy can be appropriate and useful for malaria control in some specific settings, whereas in other settings such efforts are unlikely to be cost-effective [14]. To be effective, application of *Bti* and *Bs*, like any other larviciding intervention should be guided by adequate knowledge of the prevailing mosquito vectors, their ecology and the properties of the bacterial larvicide used [12, 16].

As some countries in sub-Saharan Africa are making good progress in malaria control, larviciding, particularly with bacterial larvicides, needs to be included in the list of viable options to intensify elimination campaigns. Thus, information on the effectiveness and feasibility of applying bacterial larvicides for mosquito control is important for designing and implementing larvae control operations to supplement interventions targeting adult mosquitoes. Here, we reviewed the available literature on the use of bacterial larvicides for malaria vector control in SSA in order to provide an informed background for designing and implementing larvae control using bacterial larvicides. The present review was designed to complement available literature on larval source management by reviewing only studies on bacterial larvicides used for malaria vector control and limited its scope to studies conducted in SSA.

## Methods

### Search strategy and article selection

Articles for this review were identified through PubMed, as well as from the extensive files of the authors and the reference lists of relevant articles. The PubMed search was conducted by using the following search terms: “microbial larvicide” or “*Bacillus thuringiensis israelensis*” or “*Bacillus sphaericus*”. The PubMed search resulted in 1112 articles, of which 1077 were excluded (after screening titles and abstracts) because they did not address the effect of bacterial larvicides on malaria vectors or malaria transmission, or the studies were not from the SSA region. Moreover, a total of 21 relevant articles were obtained from the files of the authors and the reference lists of identified relevant articles. Thus, a total of 56 articles were considered for full-text reading and used for this review. Operational studies with more than one published article that reported different outcome measure of interest were all included. For articles that reported the impact of bacterial larvicides on combined malaria and non-malaria vectors or of bacterial larvicides combined with other control methods, only data on malaria vectors and on control effects attributable to microbial larvicides were considered. Data from the selected articles were extracted onto a data extraction form created in Microsoft Excel to easily assess and compare information on key study aspects such as bacterial larvicide products, experimental designs, surveyed larval habitats, the feasibility of the application, the impact of larvicides, effect size, intervention costs, safety and acceptability. Due to the wide range and heterogeneity of the study designs, larvicide products tested, application rates and effect size reported, data for the laboratory, semi-field and field studies are presented separately in the results section. Studies conducted in laboratory settings using laboratory colonized malaria vectors were classified as “laboratory studies” whereas those conducted in simulated field conditions (artificial larval habitats set in open fields) with field collected or laboratory reared mosquitoes were categorized as “semi-field studies”. “Field studies” included trials against natural vector populations in natural breeding habitats of malaria vectors.

## Results

### Description of the reviewed studies

**Fig. 1:**
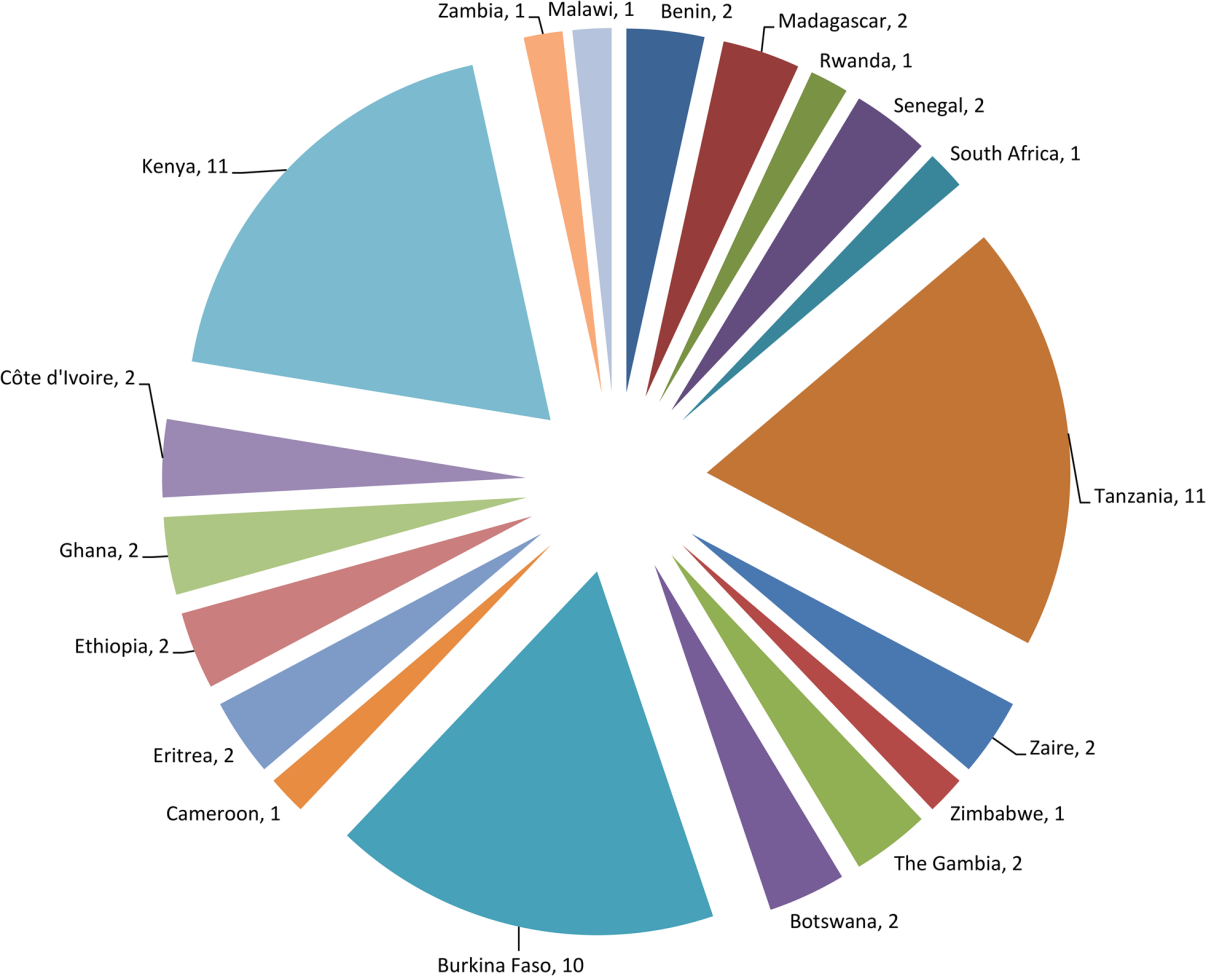
Number of reviewed publications by country. For publications involving multi-country studies, each country was counted towards the total, e.g. Kenya and Tanzania [94] and Botswana and Zimbabwe [79]. *Note*: Zaire: now The Democratic Republic of the Congo

A total of 56 studies were reviewed. More than half (*n* = 32, 57.1%) were conducted in three countries, namely Kenya (*n* = 11, 19.6%), Tanzania (*n* = 11, 19.6%) and Burkina Faso (*n* = 10, 17.9%) (Fig. 1). The articles were published from 1987 to 2019 and represented over 3 decades of testing of bacterial larvicides in SSA. Of the 56 reviewed articles, 8 (14.3%) had non-interventional components, dealing mainly with community acceptability and/or cost analysis of larvicide interventions. Of the remaining 48 articles, 3, 3 and 32 reported studies that evaluated the activity of larvicides in laboratory, semi-field and field settings only, respectively, whereas 10 articles reported a mixture of these types of studies (Table 1).

**Table 1:** Overview of the reviewed articles reporting on bacterial larvicides tested in sub-Saharan Africa, and on the type of studies they describe

Study type^a^	No. of articles	References
Laboratory only	3	[21, 64, 65]
Laboratory + semi-field	3	[66–68]
Laboratory + field	1	[26]
Laboratory + semi-field + field	3	[20, 69, 70]
Semi-field only	3	[71–73]
Semi-field + field	3	[47, 74, 75]
Field only	32	[10, 18, 19, 22–25, 27, 30, 32, 48–51, 60, 61, 76–91]
Non-interventional	8	[28, 29, 31, 52, 92–95]
Total	56	

^a^A total of 3 laboratory studies, 3 semi-field studies, 32 field studies, 10 mixed study types and 8 non-interventional studies were reported in the 56 reviewed articles

Of the 39 field studies that involved larvicide application, 24 (61.5%) were conducted in rural settings, whereas 15 (38.5%) were conducted in urban or peri-urban settings (Table 2). Four of these (10.3%) reported on two large-scale larvae control operations conducted in western Kenya and Dar es Salaam, Tanzania. A variety of *Anopheles* larval habitats were reported in the reviewed articles and these differed considerably within the trial sites. Application of bacterial larvicides in the field studies targeted *An. gambiae* (*s.l.*) and *An. funestus*, the major malaria vectors in SSA (Table 2).

**Table 2:** Reviewed field studies on bacterial larvicides tested in sub-Saharan Africa, including information on study areas, survey periods, larval habitats and their associated malaria vectors

Study site	Settings	Survey period	Malaria vector	Habitat types	References
Highland, Madagascar	Rural	Jan 1991–Mar 1992; Jan 1993–Mar 1994	*An. arabiensis*	Pools, ditches, rice fields	[25, 82]
Cove, Benin	Rural	Nov 2011–Dec 2011	*An. gambiae*	Rice fields	[75]
Mbita, Kenya	Rural	Jun 2002–Sep 2004	*An. gambiae* (*s.l.)*; *An. funestus*	Rock pools, ditches, puddles, swamps, cement lined pits, burrow pits, footprints, tyre tracks	[49]^a^
Vihiga and Kericho, Kenya	Rural	Apr 2008–Mar 2009	*An. gambiae* (*s*.*l*.)	Pools, ditches, hoof prints, erosion pits	[76]
Floodplains of the River Gambia, Gambia	Rural	Aug 2005–Nov 2005; May 2006–Nov 2007	*An. gambiae* (*s*.*l*.)	Rice fields, pools, flood water areas, swamps	[50, 69]
Muheza, Tanzania	Rural	Oct 1990–Dec 1990	*An. funestus*	Pools in blocked streams	[26]
Mvomero, Tanzania	Rural	May 2006–Jul 2008; Mar 2012–May 2013	*An. gambiae* (*s*.*l*.)*; An. funestus*	Ditches, rice fields, puddles, road side canals, swamps, river/stream bed pools, ponds, wetlands, cement lined tanks, streams, wells	[23, 87]
Kotiokh, Senegal	Rural	Not stated	*An. gambiae* (*s*.*l*.)	Ponds	[83]
Vihiga and Kakamega, Kenya	Rural	Jul 2005–Jan 2007; May 2010–Oct 2010; Feb 2011–Apr 2011; Jan 2016–May 2016	*An. gambiae* (*s*.*l*.); *An. funestus*	Drainage canals, abandoned gold mines, ponds, fish ponds, road side canals	[10, 24, 47, 48, 86]
Tiémélékro, Côte d’Ivoire	Rural	Dec 2005–Jul 2006	*An. gambiae* (*s*.*l*.); *An. funestus*	Not specified	[85]
Bobirwa, Botswana; Buhera, Zimbabwe	Rural	Jun 2015–Sep 2015; Aug 2012–Oct 2013	*An. arabiensis*	Riverbed pools/drains, hoof prints	[79, 81]
Anseba, Gash-Barka, Debub and North Red Sea zones, Eritrea	Rural	Not stated	*An. arabiensis*	Pools, canals, swamps, dams, stream bed pools, wells, ponds	[88, 89]
Chikhwawa, Malawi	Rural	May 2016–April 2018	Not stated	Not stated	[90]
Nouna, Burkina Faso	Rural	2013–2015	*An. gambiae* (*s*.*l*.); *An. funestus*	Rice fields, ponds, brickworks	[61]
Maroua, Cameroon	Urban	Feb 1992–Nov 1993	*An. gambiae* (*s*.*l*.)	Ditches, puddles	[18]
Cotonou, Benin	Urban	May 2007–Jul 2008	*Anopheles* spp.	Pools	[22]
Dar es Salaam, Tanzania	Urban	Mar 2006–Apr 2014	*An. gambiae* (*s*.*l*.)	Ditches, rice paddies, puddles, swamps, water storage containers, construction pits, ponds, riverbeds, habitats associated with urban agriculture, drains, mangrove swamps	[32, 60, 91]^a^
Kinshasa, Zaire^b^	Urban	Oct 1989–Jan 1990; May 1991–Sep 1991	*An. gambiae* (*s*.*l*.)	Irrigation ponds, rice fields, swamps	[51, 78]
Bobo-Dioulasso, Burkina Faso	Urban	Jul 1985–Sep 1985;	*An. gambiae* (*s*.*l*.)	Ponds	[20]
Ouagadougou, Burkina Faso	Urban	Jul 1984–Aug 1984; 1996–1997	*An. gambiae* (*s*.*l*.)	Pools, rain puddles	[27, 70]
Ouagadougou and Bobo-Dioulasso, Burkina Faso	Urban	Jul 1999–Oct 2000	*An. gambiae* (*s*.*l*.)	Rain puddles	[84]
Dakar, Senegal	Urban	Jan 2012–Dec 2012	*An. gambiae* (*s*.*l*.)	Rain puddles	[74]
Malindi, Kenya	Urban	Jun 2006–Dec 2007	*An. gambiae* (*s*.*l*.)	Unused swimming pools, drainage, puddles, swamps, water tank/trough, wells, ponds, manholes, car tracks, cesspits	[77, 80]
Lusaka, Zambia	Urban	June 2011–Aug 2011	*An. gambiae* (*s*.*l*.); *An. funestus*	Dams, marshes, ponds, streams	[19]
Kilosa, Tanzania	Rural	Feb 2016–Mar 2016	*–*	Rice fields	[30]

*Note*: All studies were operational research in design except studies marked with ^“a”^ were large-scale control programmes

^b^Zaire: now The Democratic Republic of the Congo

### Bacterial larvicide products evaluated

An overview of the evaluated bacterial larvicide products in the reviewed studies is shown in Table 3. Five studies did not specify *Bti* and/or *Bs* products evaluated [18–22]. Of the reviewed field studies that reported larvicide application, 13 (33.3%) tested *Bti* and *Bs* concurrently (in alternating fashion or in separate larval habitats in the same study site) whereas 13 (33.3%) and 7 (17.9%), respectively, tested only *Bti* or *Bs*. Six (15.4%) studies tested larvicide products that were formulated with a combination of both *Bti* and *Bs* toxins, namely FourStar® briquettes, VectoMax® corn granules (CG) and Culinexcombi® tablets. Water-dispersible granules (WDG) and CG formulations of the commercial strains of *Bti* were the majority of larvicide products tested in the reviewed studies, reported by 19 and 11 studies, respectively. In the reviewed studies, application rates for the bacterial larvicide products varied considerably with a strain of the bacterium (*Bti* or *Bs*), product formulation and inherent potency of the product as measured by their toxicity in international toxic units per milligram (ITU/mg).

**Table 3:** Commercial bacterial larvicide products based on *Bacillus thuringiensis* var. *israelensis* and *Bacillus sphaericus* applied in the reviewed studies from sub-Saharan Africa

Product (formulation)	Bacterial strain (potency)	Manufacturer	Application rate	References
VectoBac® 12 (AS)	*Bti* (200–1200 ITU/mg)	Abbott Laboratories, North Chicago, IL, USA; Bayer AG, Leverkusen, Germany	0.3–6.0 l/ha	[78, 81, 82]
VectoBac® (G)	*Bti* (200 ITU/mg)	Abbott Laboratories, North Chicago, IL, USA; Bayer AG, Leverkusen, Germany; Valent Biosciences, Corp, IL, USA	2.0–20.0 kg/ha	[10, 74, 75, 78, 81, 82, 88, 89]
VectoBac® (WDG)	*Bti* (2700–3000 ITU/mg)	Valent Biosciences, Corp, IL, USA	0.2–2.0 kg/ha	[10, 29, 32, 49, 50, 52, 60, 61, 67–69, 71, 72, 74, 76, 79, 85, 90, 94]
VectoBac® (CG)	*Bti* (200 ITU/mg)	Valent Biosciences, Corp, IL, USA	4.0–10.0 kg/ha	[28, 30, 32, 49, 50, 60, 69, 77, 87, 91–94]
VectoBac DT (Tab)	*Bti* (1.3 × 10^6^ ITU *Bti*)	Valent Biosciences, Corp, IL, USA	1 tablet/2000 l	[77]
Teknar HP-D (LC)	*Bti* (1500 ITU/mg)	Sandoz, USA	1.25 l/ha	[25, 70]
Bactimos® (WP)	*Bti* (3500 ITU/mg)	Biochem Products, Moutchanin, USA	0.25–0.5 kg/ha	[70]
VectoBac® (WP)	*Bti* (200 ITU/mg)	Abbott Laboratories, North Chicago, IL, USA	0.5–1.0 kg/ha	[70]
Bactivec® (WBS)	*Bti* (Not specified)	Labiofam AS, Havana, Cuba	5 ml/l	[23, 91]
Bactimos® (PP)	*Bti* (10000 ITU/mg)	Valent Biosciences, Corp, IL, USA	0.09 kg/ha	[67]
ABG6138G	*Bti* (200 ITU/mg)	Abbott Laboratories, North Chicago, IL, USA	2.8–5.6 kg/ha	[70]
IPS-82	*Bti* (not specified)	Institute Pasteur, Paris, France	na	[65]
HD-522	*Bti* (not specified)	*Bacillus* Genetic Stock Center, Ohio State University, USA	na	[64]
VectoLex® (WDG)	*Bs* (650 ITU/mg)	Valent Biosciences, Corp, IL, USA	1.0–10.0 kg/ha	[10, 49, 60, 66, 67, 69, 84, 85]
VectoLex® (CG)	*Bs* (50–670 ITU/mg)	Valent Biosciences, Corp, IL, USA	11.2–30.0 kg/ha	[49, 60, 69, 88, 89]
Spherimos (Briquets)	*Bs* (Not specified)	Summitt Chemical Co. Baltimore, MD, USA	1 briquet/m^2^	[26]
Spherimos (G)	*Bs* (Not specified)	Abbott Laboratories, North Chicago, IL, USA	30.0 kg/ha	[83]
VectoLex® (G)	*Bs* (20–200 ITU/mg)	Abbott Laboratories, North Chicago, IL, USA	10.0–30.0 kg/ha	[10, 51, 78]
Spherimos (FC)	*Bs* (Not specified)	Abbott Laboratories, North Chicago, IL, USA; Duphar BV, Weesp, Netherlands	30.0–60.0 kg/ha	[26, 83]
Griselesf® (WBS)	*Bs* (Not specified)	Labiofam AS, Havana, Cuba	10ml/l	[23]
ABG6185G	*Bs* (5 × 10^10^ spores/g)	Abbott Laboratories, North Chicago, IL, USA	2.0–8.0 kg/ha	[82]
SPH-88	*Bs* (not specified)	Institute Pasteur, Paris, France	na	[65]
*Bs* TP	*Bs* (1600 *Bs* ITU/mg)	Valent Biosciences, Corp, IL, USA	na	[67]
VectoMax® (CG)	*Bti* + *Bs* (52 ITU/mg *Bti* and 50 ITU/mg *Bs*)	Valent Biosciences, Corp, IL, USA	7.5 kg/ha	[74, 86]
Culinexcombi (Tab)	*Bti* + *Bs* (1.0 × 10^6^ ITU *Bti* + 2.5 × 10^4^ ITU *Bs)*	Culinex GmbH, Germany	1 tablet/2000 l	[77, 80]
FourStar® (Briquettes)	*Bti* + *Bs* (Not specified)	Adapco Inc. Sanford, FL, USA; Central Life Sciences, Sag Harbor, NY, USA	1 briquette/100 ft^2^	[24, 47, 48]
BTBSWAX (Wax)	*Bti* + *Bs* (Not specified)	ISCA Technologies, Riverside, CA, USA	1–2 g/m^2^	[73]

*Notes*: Non-commercial formulations tested included: locally made slow release granular formulation of *Bti*/*Bs* [27, 95]. *Bs* isolate 1593 and 2362 [70] and LL3 [24, 48]. Reported bacterial strains: *Bti*: AM-6552, IPS-82, HD-522 and BMP 144. *Bs:* 2362, ABG 6185 and SPH-88. Serotypes: *Bti*; H-14; *Bs*; H5a5b

*Abbreviations*: *Bti*, *Bacillus thuringiensis* var*. israelensis*; *Bs*, *Bacillus sphaericus*; AS, aqueous suspension; G, granules; WDG, water-dispersible granules; CG, corn granules; Tab, tablets; LC, liquid concentrate; WP, wettable powder; WBS, water-based suspension; PP, primary powder; TP, technical powder; FC, flowable concentrate; ITU, international toxic units; na, not applicable

### The activity of *Bti* and *Bs* in laboratory settings

Ten (17.9%) of the reviewed articles presented the findings of the efficacy of *Bti* and *Bs* on *An. gambiae* (*s.l.*) and *An. funestus* in laboratory settings (Table 4). The bio-potency of *Bti* and *Bs* based products varied between 1500–10,000 *Bti* ITU/mg and 650–1600 *Bs* ITU/mg, respectively. In most cases, laboratory experiments were conducted for 24 and 48 h for *Bti* and *Bs*, respectively. For *Bti*, the lethal concentration value that caused 50 and 90/95% mortality of *An. gambiae* (*s.l.*) larvae (LC_50_ and LC_90/95_) ranged between 0.006–0.662 mg/l and 0.132–1.743 mg/l, respectively. For *Bs*, the LC_50_ and LC_90_/_95_ values for the same mosquito species ranged between 0.002–0.342 mg/l and 0.018–1.807 mg/l, respectively. For *An. funestus*, LC_50_ and LC_95_ values after 48 h of exposure to *Bs* were 1.0 mg/l and 6.0 mg/l, respectively. For studies that reported the potency in spores per milliliter, LC_50_ and LC_90_ values after 24 h of exposure to *Bti* in different malaria vectors are presented in Table 4.

**Table 4:** Laboratory trials using *Bacillus thuringiensis* var. *israelensis* and *Bacillus sphaericus* against malaria vectors in sub-Saharan Africa

Site (Country)	Bacterial strain (potency)	Product (formulations)	Species tested (larval instar)	Exposure time (h)	LC_50_	LC_90/95_	References
ICIPE (Kenya)	*Bti* (2700 ITU/mg)	VectoBac® (WDG)	*An. gambiae* (*s*.*s*.) (3rd)	24	0.021	0.201	[67]
	*Bs* (650 BsITU/mg)	VectoLex® (WDG)	*An. gambiae* (*s*.*s.*) (3rd)	24	0.004	0.038	
	*Bti* (10000 ITU/mg)	Bactimos® (PP)	*An. gambiae* (*s*.*s.*) (3rd)	24	0.006	0.090	
	*Bs* (1600 ITU/mg)	(TP)	*An. gambiae* (*s*.*s*.) (3rd)	24	0.002	0.018	
Ouagadougou (Burkina Faso)	*Bti* (3500 ITU/mg)	Bactimos® (WP)	*An. gambiae* (*s*.*l.*) (3rd and 4th)	24	0.081	0.231^a^	[70]
	*Bti* (2000 ITU/mg)	VectoBac® (WP)	*An. gambiae* (*s*.*l*.) (3rd and 4th)	24	0.110	0.375^a^	
	*Bti* (1500 ITU/mg)	Teknar (FC)	*An. gambiae* (*s*.*l.*) (3rd and 4th)	24	0.662	1.743^a^	
	*Bs* (not stated)	1593 IF-119 (SD)	*An. gambiae* (*s*.*l.*) (3rd and 4th)	48	0.043	0.107^a^	
	*Bs* (not stated)	2362 IF-118 (SD)	*An. gambiae* (s.l.) (3rd and 4th)	48	0.022	0.130^a^	
MRC, Farafenni (Gambia)	*Bs* (650 *Bs* ITU/mg)	VectoLex® (WDG)	*An. gambiae* (*s*.*s.*) (3rd)	24	0.004	0.023	[69]
	*Bti* (3000 ITU/mg)	VectoBac® (WDG)	*An. gambiae* (*s*.*s.*) (3rd)	24	0.039	0.132	
KEMRI, Kisumu (Kenya)	*Bti* (7000 ITU/mg)	Not stated	*An. gambiae* (*s*.*l*.) (3rd)	24	0.062	0.797	[21]
	*Bs* (1000 ITU/mg)	Not stated	*An. gambiae* (*s*.*l*.) (3rd)	48	0.058	0.451	
KCCR, Kumasi (Ghana)	*Bti* (3000 ITU/mg)	VectoBac® (WDG)	*An. gambiae* (3rd)	24	0.026	0.136	[68]
Bobo-Dioulasso (Burkina Faso)	*Bs* (not stated)	(FC)	*An. gambiae* (*s*.*s*.) (3rd)	48	0.342	1.807	[20]
UFS, Muheza (Tanzania)	*Bs* (not stated)	Spherimos (FC)	*An. funestus* (4th)	48	1.0	6.0	[26]
KCCR, Kumasi (Ghana)	*Bs* (not stated)	VectoLex® (WDG)	*An. gambiae* (*s*.*l.*) (3rd and 4th)	24	0.0027	0.0086	[66]
NOCMVD, Nazareth (Ethiopia)	*Bti* (not stated)	IPS-82	*An. arabiensis* (3rd)	48	0.0018^b^	–	[65]
	*Bs* (not stated)	SPH-88	*An. arabiensis* (3rd)	48	0.0076^b^	–	
NICD, Johannesburg (South Africa)	*Bti* (not stated)	Not stated	*An. quadriannulatus* (3rd)	24	2.97	5.02	[64]
		–	*An. arabiensis* (3rd)	24	3.72	10.10^a^	
		–	*An. gambiae* (3rd)	24	3.76	7.70^a^	
		–	*An. merus* (3rd)	24	3.82	6.65^a^	
		–	*An. funestus* (3rd)	24	4.44	13.50^a^	

^a^LC_90/95_: a dose marked with ^a^ represents LC_90_; unmarked doses represent LC_95_

^b^Doses provided in µg/l were converted to mg/l for a uniform presentation. Concentrations reported in mg/l except for study [64] are given in 10^4^ spores/ml

*Abbreviations*: *Bti*, *Bacillus thuringiensis* var. *israelensis*; *Bs*, *Bacillus sphaericus*; ITU, international toxic units; WDG, water-dispersible granules; PP, primary powder; TP, technical powder; FC, flowable concentrate; SD, spray dried; ICIPE, International Centre of Insect Physiology and Ecology; MRC, Medical Research Council; KCCR, Kumasi Centre for Collaborative Research; UFS, Ubwari Field Station; NOCMVD, National Organization for the Control of Malaria and other Vector-borne Diseases; NICD, National Institute of Communicable Diseases; LC, lethal concentration (concentration that kills 50/95% of the test subjects)

### The activity of *Bti* and *Bs* in semi-field conditions

A total of 12 (21.4%) studies reported experiments with *Bti* and *Bs* conducted in semi-field conditions to establish their effectiveness and duration of control (Table 5). The larval habitats treated contained *Anopheles gambiae* (*s.l.*), a mixture of *Anopheles* and culicine species, *An. arabiensis* and *An. gambiae* (*s.s*.) in 5, 4, 2 and 1 of these studies, respectively. Larvicide application rates varied considerably among the studies with products based on *Bti* having relatively lower application rates compared to *Bs*. With respect to larvicide formulation, application rates for water-dispersible granules (WDG) were lower than corn granules (CG) or granules (G) due to their inherently high potency. The studies reported appreciable larval reductions in the treated larval habitats for 2 to 14 days post-treatment. Of the tested products, the highest larval reductions and the most prolonged effect was seen in studies that tested VectoMax® CG, with 98–100% reduction in late larval instars for 2 weeks. On the other hand, the pupal reductions in treated larval habitats varied between 64–100%, with residual effects ranging from 7 days to 3 months. A very high residual effect in pupal control was observed in a study that tested a slow release formulation of bacterial larvicide (FourStar®) that combined both *Bti* and *Bs* (Table 5).

**Table 5:** Semi-field trials using commercial products of *Bacillus thuringiensis* var. *israelensis* and *Bacillus sphaericus* against malaria vectors in sub-Saharan Africa

Test site (Country)	Larvicide type (potency)	Product (formulation)	Species tested	Application rate	% larval reduction^e^ (Residual)	% pupae reduction (Residual)	References
CRSN, Nouna (Burkina Faso)	*Bti* (3000 ITU/mg)	VectoBac® (WDG)	*An. gambiae* (*s*.*l.*)	0.2–1.0 mg/l	90–100 (4 days)	98.5 (21 days)	[71]
CREC (Benin)	*Bti* (200 ITU/mg)	VectoBac® (GR)	*An. gambiae*	0.6–1.2 g/m^2^	–	> 80 (19 days)	[75]
MRC, Farafenni (Gambia)	*Bti* (3000 ITU/mg)	VectoBac® (WDG)	*Anopheles* and Culicinae^a^	0.2 kg/ha	81–100 (5 days)	64–94 (7 days)	[69]
	*Bs* (650 ITU/mg)	VectoLex® (WDG)	*Anopheles* and Culicinae^a^	0.5–5.0 kg/ha	96–100 (1–2 days)	> 95 (7 days)	
KCCR, Kumasi (Ghana)	*Bti* (not stated)	VectoBac® (WDG)	*Anopheles* and Culicinae^b^	0.2–0.4 mg/l	51–100 (4 days)	–	[68]
Bobo-Dioulasso (Burkina Faso)	*Bs* (not stated)	2362 (FC)	*An. gambiae* (*s*.*l*.)	0.1–10.0 g/m^2^	100 (7–10 days)	92 (3–10 days)	[20]
KCCR,Kumasi (Ghana)	*Bs* (not stated)	VectoLex® (WDG)	*Anopheles* and Culicinae^c^	0.5–1.0 mg/l	70–100 (10 days)	100 (12 days)	[66]
ICIPE, Suba (Kenya)	*Bti* (2700 ITU/mg)	VectoBac® (WDG)	*Anopheles* and Culicinae^d^	0.2–1.6 mg/l	88–100 (4 days)	95	[67]
	*Bs* (650 Bs ITU/mg)	VectoLex® (WDG)	*Anopheles* and Culicinae^d^	1 and 5 mg/l	100 (11 days)	100 (14 days)	
Kisian, Kisumu (Kenya)	*Bti* + *Bs* (not stated)	FourStar® (Briquettes)	*An. gambiae* (*s*.*l*.)	1 briquette per 9.3 m^2^	–	85.5 (180 days)	[47]
Ouagadougou (Burkina Faso)	*Bs* (not stated)	1593 IF-119 (Spray-dried)	*An. gambiae* (*s*.*l*.)	0.12–0.24 kg/ha	95.8–100 (2 days)	–	[70]
	*Bs* (not stated)	2362 IF-118 (spray dried)	*An. gambiae* (*s*.*l*.)	0.12–0.24 kg/ha	100 (2 days)	–	
Tolay (Ethiopia)	*Bti* (3000 ITU/mg)	VectoBac® (WDG)	*An. arabiensis*	0.05–0.2 g/m^2^	50–100 (13 days)	–	[72]
DP, Dakar (Senegal)	*Bti* (3000 ITU/mg)	VectoBac® (WDG)	*An. arabiensis*	0.03 g/m^2^	94–100 (14 days)	–	[74]
	*Bti* (200 ITU/mg)	VectoBac® (GR)	*An. arabiensis*	0.5 g/m^2^	92–100 (14 days)	–	
	*Bti*/*Bs* (52/50 ITU/mg)	VectoMax® (CG)	*An. arabiensis*	0.75 g/m^2^	98–100 (14 days)	–	
Bouake (Côte d’Ivoire)	*Bti* + *Bs* (Not stated)	BTBSWAX® (Wax)	*An. gambiae* (*s*.*s*.)	1–2 g/m^2^	–	< 10– > 80 (10 days)	[73]

*Note*: Proportional of tested *Anopheles:* ^a^*Anopheles* population accounted for 40%

^b^*Anopheles* population accounted for 87%

^c^*Anopheles* population accounted for 89%

^d^*Anopheles* population accounted for 85%

^e^Larval reductions: based on late instars except study [66] in which reductions were based on all larval instars

*Abbreviations*: CRSN, Centre de Recherche en Sante de Nouna; ITU, international toxic units; CREC, Centre de Recherche Entomologique de Cotonou; WDG, water-dispersible granules; GR, granules; FC, flowable concentrate; *Bti*, *Bacillus thuringiensis* var*. israelensis*; *Bs*, *Bacillus sphaericus*; MRC, Medical Research Council; KCCR, Kumasi Centre for Collaborative Research; ICIPE, International Centre of Insect Physiology and Ecology; DP, Department of Pikine

### The activity of *Bti* and *Bs* on immature and adult mosquitoes in field conditions

A total of 39 (69.6%) reviewed studies evaluated the activity of *Bti* and/or *Bs* in field conditions (Table 2). Of these, seven commenced the evaluations of *Bti* and/or *Bs* from the laboratory and/or semi-field conditions. Five bacterial larvicide products evaluated in the semi-field trials were also tested in the field conditions (Table 6). WDG and CG formulations of *Bt*i and *Bs* were the majority of the evaluated products, with VectoBac® WDG tested in 14 of the field studies. Reported larval reductions varied considerably with the test site, larvicide product applied and application rate. Overall, larval reductions ranging from 47 to 100% were recorded, with the residual effect lasting for 2 to 28 days following single or repeated applications of the larvicide. Substantial pupal reductions were also reported, with the most marked impact observed with FourStar®, a slow release bacterial larvicide formulation (Table 6). The least larval reductions were recorded with Bactimos® and VectoBac® wettable powder (WP) when applied once in rain pools. In the reviewed articles, it was not possible to analyze the difference in susceptibility between *An. gambiae* (*s.l.*) and *An. funestus* or variation in different ecological settings due to heterogeneity in testing conditions, products used and pooling of mosquito species data in some studies. However, the reviewed laboratory studies indicated that *An. gambiae* (*s.l.*) were more sensitive to *Bti* and *Bs* than *An. funestus* (Table 4). On the other hand, a total of 14 (35.9%) reviewed field studies reported the activity of *Bti* and/or *Bs* in adult mosquitoes and/or malaria transmission. Different levels of reductions in adult mosquitoes and/or malaria transmission were reported with single or repeated applications of *Bti* and/or *Bs* (Table 7).

**Table 6:** Field trials of commercial products of *Bacillus thuringiensis* var. *israelensis* and *Bacillus sphaericus* against immature stages of malaria vectors in sub-Saharan Africa

Country	Vector targeted	Product (Potency)	Application rate	Application cycle	% Larval reduction	Residual effect	References
Mahitsy, Madagascar	*An. arabiensis*	VectoBac® 12 AS (1200 ITU/mg)	0.3–1.0 l/ha	Once	89–100^a^	2 days	[82]
		VectoBac® G (200 ITU/mg)	2.0–10.0 kg/ha	Once	67–100^a^	2 days	
		ABG6185 (G)	2.0–18.0 kg/ha	Once	37–100^a^	2 days	
Mbita, Kenya	*An. gambiae* (*s*.*l*.); *An. funestus*	VectoBac® WDG (3000 ITU/mg) / VectoBac® CG (200 ITU/mg)	0.2 kg/ha / 5.0 kg/ha	Variable^b^	99	7 days	[49]
		VectoLex® WDG (650 ITU/mg) / VectoLex® CG (50 ITU/mg)	1.0 kg/ha / 15.0 kg/ha	2 weeks	99	23 days	
Dar es Salaam, Tanzania	*An. gambiae* (*s*.*l*.)	VectoBac® WDG (3000 ITU/mg)/ VectoBac® CG (200 ITU/mg)	0.4 kg/ha / 10.0 kg/ha	1 week	96^a^	7 days	[32, 60]
		VectoLex® WDG (650 ITU/mg)/ VectoLex® CG (50 ITU/mg)	2.0 kg/ha / 30.0 kg/ha	1 week	96^a^	7 days	
Kakamega and Vihiga, Kenya	*An. gambiae* (*s*.*l*.); *An. funestus*	VectoBac® WDG/G; VectoLex® WDG/G	–	1 week	91.1	7 days	[10]
Malindi, Kenya	*An. gambiae* (*s*.*l*.)	VectoBac DT	1 tab/2000 l	Once	89–99	8 days	[77]
		Culinexcombi Tab (1.0 × 10^6^ ITU Bti + 2.5 × 10^4^ ITU *Bs*)	1 tab/2000 l	Once	77–100	8 days	
Kinshasa, Zaire^g^	*An. gambiae* (*s*.*l*.)	VectoBac® G (200 ITU/mg)	10.0–0.0 l/ha	Once	86–100^a^	2 days	[78]
		VectoLex® G (200 ITU/mg)	10.0–30.0 l/ha	Once	95–100^a^	2 days	
Floodplains of the River Gambia, Gambia	*Anopheles* sp.	VectoBac® WDG 3000 ITU/mg)/ VectoBac® CG (200 ITU/mg)	0.2 kg/ha / 4.0 kg/ha	1 week	100	7 days	[69]
		VectoLex® WDG (650 ITU/mg)	1.0 kg/ha	1 week	100	2 days	
Floodplains of the River Gambia, Gambia	*An. gambiae* (*s*.*l*.)	VectoBac® WDG (3000 ITU/mg)/ VectoBac® CG (200 ITU/mg)	0.2 kg/ha / 5.0 kg/ha	1 week	88^a^	7 days	[50]
Bobirwa, Botswana; Buhera, Zimbabwe	*Anopheles* sp.	VectoBac® WDG	0.3 kg/ha	2 weeks	47–95^a^	14 days	[79]
Bobirwa, Botswana	*An. arabiensis*	VectoBac® 12 AS (1200 ITU/mg)/ VectoBac® G (200 ITU/mg)	2.0 l/ha / 2 g/m^2^	Once	81–97	2 days	[81]
Anseba, Gash-Barka, Debub and North Red Sea zones, Eritrea	*An. arabiensis*	VectoBac® G (200 ITU/mg)	5.6–11.2 kg/ha	Once	54–100^a^	14–21 days	[88, 89]
		VectoLex® CG (670 ITU/mg)	11.2–22.4 kg/ha	Once	73.8–100^a^	14–21 days	
Anjiro, Madagascar	*An. gambiae* (*s*.*s.*)	Teknar HP–D LC	1.25 l/ha	Once	95.3–100^a^	3 days	[25]
Dakar, Senegal	*An. gambiae* (*s*.*l*.)	VectoBac® WDG (3000 ITU/mg)/ VectoBac® G (200 ITU/mg)/ VectoMax® CG (*Bti* 52ITU/mg and 50 *Bs* ITU/mg)	0.05 g/m^2^ / 0.5 g/m^2^ / 0.75 g/m^2^	Once	100^a^	3 days	[74]
Ouagadougou, Burkina Faso	*An. gambiae* (*s*.*l*.)	Bactimos® WP (3500 ITU/mg)	0.25–0.5 kg/ha	Once	82–95 ^a^	1 day	[70]
	*An. gambiae* (*s*.*l*.)	VectoBac® WP (2000 ITU/mg)	0.5–1.0 kg/ha	Once	86–95 ^a^	1 day	
Mvomero, Tanzania	*An. gambiae* (*s*.*l*.); *An. funestus*	Bactivec® WBS	5 ml/l	Variable^c^	79.3–98^a^	14 days	[23]
		Griselesf® WBS	10 ml/l	Variable^c^	47–76.6^a^	14 days	
Kinshasa, Zaire^g^	*An. gambiae* (*s*.*l*.)	VectoLex® G (200 ITU/mg)	10.0 kg/ha	15 days	98^a^	2 days	[51]
Bobo-Dioulasso, Burkina Faso	*An. gambiae*	Spherimos FC	0.1–10.0 g/m^2^	Once	100	1–10 days	[20]
Muheza, Tanzania	*An. funestus*	Spherimos FC	60 mg/l	Once	100 ^a^	28 days	[26]
Kotiokh, Senegal	*An. gambiae* (*s*.*l*.)	Spherimos FC	3 g/m^2^	Once	95–100^a^	5 days	[83]
		Spherimos G	3 g/m^2^	Once	100^a^	15 days	
Western, Kenya	*An. gambiae* (*s*.*l*.); *An. funestus*	VectoMax® CG	–	4 weeks	~100^a^	10 days	[86]
Cove, Benin^d^	*An. gambiae* (*s*.*l*.)	VectoBac® G (200 ITU/mg)	1.2 g/m^2^	Once	73–95	3 days	[75]
Malindi, Kenya	*An. gambiae* (*s*.*l*.)	Culinexcombi Tablets (1.0 x 10^6^ ITU Bti + 2.5 × 10^4^ ITU Bs)	1 tab/2000 l	2 weeks	99–100	10 days	[80]
Western Kenya, Kenya^e^	*An. gambiae* (*s*.*l*.); *An. funestus*	FourStar® Briquettes (not stated)	1 briq/100ft^2^	Once	–	5 months	[47]
Vihiga and Kakamega, Kenya	*An. gambiae* s.l.; *An. funestus*	FourStar®Briquettes (not stated)	1 briq/100ft^2^	Once	80^a^	4 weeks	[48]
Chikhwawa, Malawi	Not stated	VectoBac®WDG (not stated)	Not stated	Not stated	Not stated	Not stated	[90]
Lusaka, Zambia	*An. gambiae* (*s*.*l*.); *An. funestus*	Not stated	5 ml/m^2^	Not stated	Not stated	Not stated	[19]
Ouagadougou, Burkina Faso	*An. gambiae* (*s*.*l*.)	Granular *Bs* (1520 ITU/mg) / Spherimos FC (300 ITU/mg)	3.0 ml / 3.0 g/m^2^	Once	60–97	10 days	[27]
Ouagadougou and Bobo-Dioulasso, Burkina Faso	*An. gambiae* (*s*.*l*.)	Spherimos FC (80 ITU/mg) / VectoLex WSM (100% TP)	30 g/m^2^ / 0.5 g/m^2^	1 week	Not stated	Not stated	[84]
Maroua, Cameroon	*An. gambiae* (*s*.*l*.); *An. funestus*; *An. pharoensis*	*Bs* strain 2362 (not specified)	10 g/m^2^	3 rounds^f^	Not stated	Not stated	[18]
Tiémélékro, Côte d’Ivoire	*An. gambiae* (*s*.*l*.); *An. funestus* (*s*.*l*.)	VectoBac® WDG (3000 ITU/mg) / VectoLex® WDG (650 ITU/mg)	0.8 mg/l / 10 mg/l	~3 weeks	Reduced to zero	21 days	[85]
Vihiga and Kericho, Kenya	*An. gambiae* (*s*.*l*.); *An. funestus*	VectoBac® WDG (not stated)	200 g/ha	1 week	91	7 days	[76]
Cotonou, Benin	*Anopheles*	*Bti* (not stated)	50 mg/l	2 weeks	Significantly reduced	9 days	[22]
Mvomero, Tanzania	*An. gambiae* (*s*.*l*.); *An. funestus*	VectoBac® CG (ITU/mg)/	10 kg/ha	1 week	Not stated	Not stated	[87]
Nouna, Burkina Faso	*An. gambiae* (*s*.*l*.); *An. funestus*	VectoBac® WDG (not stated)	Not stated	10 days	Not stated	Not stated	[61]
Kilosa, Tanzania	Not stated	VectoBac® CG	Not stated	Not stated	Not stated	Not stated	[30]

^a^Larval reductions based on all larval instars; unmarked are based on late instars only

^b^Weekly application cycles for the first four rounds after which re-treatment was conducted when late instars were noted

^c^Larval habitats were re-treated when late instars were detected during weekly monitoring

^d^Pupal reduction reported 100% for up to 3 days [75]

^e^Pupal reduction reported 87.4–95.4% for up to 5 months [47]

^f^Three rounds of applications between March 1992–Nov 1993

^g^Zaire: now The Democratic Republic of the Congo

*Abbreviations*: ITU, international toxic units; AS, aqueous suspension; G, granules; WDG, water-dispersible granules; CG, corn granules; LC, liquid concentrate; WP, wettable powder, WBS, water-based suspension; FC, flowable concentrate; WSM, water-suspendable micro-granule; *Bti*, *Bacillus thuringiensis* var*. israelensis*; *Bs*, *Bacillus sphaericus*; tab, tablets; briq, briquettes

**Table 7:** Field trials of commercial products of *Bacillus thuringiensis* var. *israelensis* and *Bacillus sphaericus* against adult malaria vectors and the effect on malaria transmission in sub-Saharan Africa

Country	Vectors targeted	Product (application rate)	Application cycle	Percentage reduction			References
				Adult density/biting	Transmission/EIR	Malaria prevalence	
Mbita, Kenya	*An. gambiae* (*s*.*l*.); *An. funestus*	VectoBac® WDG (0.2 kg/ha); VectoBac® CG (5.0 kg/ha); VectoLex® WDG (1.0 kg/ha) and VectoLex® CG (15.0 kg/ha)	1/2 week^e^	92.0^a^	–	–	[49]
Dar es Salaam, Tanzania	*An. gambiae* (*s*.*l*.)	VectoBac® WDG (0.4 kg/ha); VectoBac® CG (10.0 kg/ha); VectoLex® WDG (2.0 kg/ha); VectoLex® CG (30.0 kg/ha)	1 week	31.3^a^	71^c^	40.0	[60]
		VectoBac® (Not stated); Bactivec® (not stated)	–	Reduced^b^	–	Reduced	[91]
Kakamega and Vihiga, Kenya	*An. gambiae* (*s*.*l*.); *An. funestus*	VectoBac® WDG; VectoBac® CG; VectoLex® WDG; VectoLex® CG	1 week	85.9^b^	73.1^d^	–	[10]
Dar es Salaam, Tanzania	*An. gambiae* (*s*.*l*.); *An. funestus*; *An. coustani*	VectoBac® WDG (0.4 kg/ha); VectoBac® CG (10.0 kg/ha)	1 week	72.0^b^	32.0^d^	Reduced	[32]
Kinshasa, Zaire^g^	*An. gambiae* (*s*.*l*.)	VectoLex® G (10.0 kg/ha)	2 weeks	13.6^a^	39.9^d,f^	–	[51]
Western, Kenya	*An. gambiae* (*s*.*l*.); *An. funestus*	FourStar® Briquettes (1 briq/100 ft^2^)	Once	60.0–85.0^b^	–	–	[47]
Nouna, Burkina Faso	*An. gambiae* (*s*.*l*.); *An. funestus*	VectoBac® WDG (not stated)	10 days	72–80^b^	–	–	[61]
Bobirwa, Botswana	*An. arabiensis*	VectoBac® 12 AS (2.0 l/ha); VectoBac® G (2.0 g/m^2^)	Once	Reduced^b^	Reduced^c^	Decreased	[81]
Eritrea	*An. arabiensis*	VectoBac® G (11.2 kg/ha); VectoLex® CG (22.4 kg/ha)	1 week	Significantly reduced^b^	–	–	[89]
Tiémélékro, Côte d’Ivoire	*An. gambiae* (*s*.*l*.); *An. funestus* (*s*.*l*.)	VectoBac® (0.8 mg/l); VectoLex® (10 mg/l)	3 weeks	Significantly reduced^a^	Significantly reduced^d^	–	[85]
Maroua, Cameroon	*An. gambiae* (*s*.*l*.); *An. funestus;*	*Bs* strain 2362 (10 g/m^2^)	3 rounds	Reduced^a^	Reduced^c^	–	[18]
Cotonou, Benin	*Anopheles*	*Bti* (50 mg/l)	2 weeks	Reduced^a^	–	Reduced	[22]
Gambia	*An. gambiae* (*s*.*l*.)	VectoBac® WDG (0.2 kg/ha); VectoBac® CG (5.0 kg/ha)	1 week	Limited^b^	Unsatisfactory^c^	No effect	[50]

Reported percentage reductions are based on: ^a^human biting; ^b^adult density; ^c^malaria transmission; and ^d^EIR

^e^1 week for Vectobac® and 2 weeks for Vectolex®

^f^Estimated from reduction in EIR from 0.238 to 0.143

^g^Zaire: now The Democratic Republic of the Congo

*Notes*: Tested product (potency): VectoBac® WDG (3000 ITU/mg); Vectobac® CG (200 ITU/mg); VectoLex® WDG (650 ITU/mg); Vectolex® CG (50 ITU/mg); VectoLex® G (200 ITU/mg)

*Abbreviations*: briq, briquettes; CG, corn granules; G, granules; ITU, international toxic units; WDG, water-dispersible granules; EIR, entomological inoculation rate

### Safety, cost effectiveness and acceptability

Five of the reviewed studies evaluated the safety of *Bti* and/or *Bs* to non-target organism co-habiting with mosquito larvae in natural larval habitats. Of these, 4 [23–26] reported that the products were fairly safe to the non-target organisms whereas the fifth [27] indicated that *Bti* caused mortalities in Psychodidae larvae. Six studies evaluated the economic costs of implementing bacterial larvicides interventions in the tropical conditions of SSA (Table 8). The costs varied greatly depending on the ecology of the vectors, the larvicide product deployed and the size of the human population covered by the intervention. The cost per person protected per year (PPPY) varied from USD 0.44 in Ouagadougou, Burkina Faso to USD 2.50 in Mbita, Kenya. The cost PPPY was relatively higher in the rural (range of USD 0.77–2.50) than in the urban settings (range of USD 0.44–0.94). Five of the reviewed studies monitored the acceptability of microbial larvicide interventions to the community members and concluded that they were highly accepted by the general community [23, 28–31]. However, challenges related to accessing larval habitats in people’s compounds were reported from the large-scale larviciding intervention conducted in Dar es Salaam, Tanzania [32]. In general, *Bti* and/or *Bs* larvicide products were perceived as relatively easy to use and suitable to apply with hand and/or conventional sprayers.

**Table 8:** Cost (in USD) of bacterial larvicide interventions for malaria control in sub-Saharan Africa

Country (costing year)	Location (settings)	Population protected	Larvicide (potency)	Product (formulation)	Targeting strategy	Average annual costs	Cost PPPY	References
Tanzania (2006)	Dar es Salaam (urban)	592338	*Bti* (200 ITU/mg)	VectoBac® (CG)	None	559476.3^d^	0.94	[94]
Kenya (2006)	Vihiga (rural)	609324	*Bti* (200 ITU/mg)	VectoBac® (CG)	Spatial and temporal^a^	916908^d^	1.50	[94]
Kenya (2006)	Vihiga (rural)	609324	*Bti* (3000 ITU/mg)	VectoBac® (WDG)	Spatial and temporal^a^	480735^d^	0.79	[94]
Kenya (2006)	Mbita (rural)	55558	*Bti* (200 ITU/mg)	VectoBac® (CG)	Spatial^b^	138866^d^	2.50	[94]
Kenya (2006)	Mbita (rural)	55558	*Bti* (3000 ITU/mg)	VectoBac® (WDG)	Spatial^b^	107669^d^	1.94	[94]
Burkina Faso (2013)	Nouna (rural)	156000	*Bti* (3000 ITU/mg)	VectoBac® (WDG)	None	163038	1.05	[52]
Burkina Faso (2013)	Nouna (rural)	156000	*Bti* (3000 ITU/mg)	VectoBac® (WDG)	Spatial^c^	120239	0.77	[52]
Tanzania (2014)	Mvomero (rural)	37083	*Bti* (not stated)	VectoBac® (CG)	None	53782.53	1.44	[93]
Tanzania (2012)	Dar es Salaam (urban)	6875784	*Bti* (200 ITU/mg)	VectoBac® (CG)	None	5111234	0.87	[92]
Kenya (2005)	Mbita (rural)	8000	*Bti* (200 and3000) + *Bs* (50 and 650 ITU/mg)	VectoBac® (WDG & CG) + VectoLex® (WDG & CG)	Spatial^b^	6773–7026	0.85–0.89	[49]
Burkina Faso (1999)	Bobo-Dioulasso (urban)	19245	*Bs* (not stated)	VectoLex® (WDG); Spherimos (FC); Locally made (SRG)	None	8400^e^	0.44	[95]
Burkina Faso (1999)	Ouagadougou (urban)	17776	*Bs* (not stated)	VectoLex® (WDG), Spherimos (FC), Locally made (SRG)	None	8400^e^	0.47	[95]

^a^Intervention in valley bottoms during the main rainy season

^b^Intervention covering two third of the populated lowlands

^c^Targeted application of 50% of the most productive habitats

^d^Mid-point published price of larvicide

^e^Costing included interventions to control both *Anopheles gambia*e and *Culex quinquefasciatus* (1€ was approximately 1 USD in the costing year)

*Abbreviations*: PPPY, per person protected per year; *Bti*, *Bacillus thuringiensis* var*. israelensis*; *Bs*, *Bacillus sphaericus*; ITU, international toxic units; CG, custom granules; WDG, water-dispersible granules; SRG, sustained-release granular; FC, fluid concentrate

## Discussion

After several years of encouraging reports on global malaria decline [33], the 2018 world malaria report indicated that no further significant progress in reducing global malaria cases was made during the 2015–2017 time frame [34]. The persisting malaria transmission occurs despite implementation in time and space of widely effective malaria control interventions, mainly anti-malarial drugs and insecticide-based vector control methods [34]. With the observed resilience in malaria transmission, the current control interventions need to be complemented with other novel methods in an integrated manner to further reduce the malaria burden. Larval source management is an important strategy in malaria control and its potential to lower malaria transmission has been well documented [8, 11, 35–37]. When integrated with adult mosquito control interventions, such as LLINs or IRS, the strategy has been found to have a complementary role in lowering malaria transmission [10]. The present article reviews the available literature on implementation of bacterial larvicides for malaria vector control in sub-Saharan Africa (SSA) to provide an informed background for designing larval source management using these agents.

Reduction in malaria burden and intensity of parasite transmission [expressed as entomological inoculation rate (EIR), a measure of infectious bite per person per unit time] recorded over the past two decades is an important epidemiological juncture to intensify malaria control measures. It has been shown that once EIRs fall below one infectious bite per person per year, malaria burden becomes much more responsive to further reductions in transmission [38]. Thus, as malaria continues to decline, larviciding interventions may have a much greater epidemiological impact as a supplementary intervention, secondary to primary front-line options like LLINs/IRS. Moreover, larviciding becomes a more feasible intervention for IVM once malaria transmission has been reduced to low and moderate levels by LLINs/IRS or once these tools have reached their maximum practical effect [14]. Recognizing the limitations of the primary front-line vector control tools and reduced progress of malaria control recorded in recent years, call for accelerated development and adoption of diverse options available for malaria vector control [39].

The reviewed studies on bacterial larviciding conducted in field conditions were carried out in typical *Anophele*s breeding habitats found in SSA and targeted the main malaria vectors *An. gambiae* (*s.l.*) and *An. funestus*. The laboratory and semi-field studies targeted the same vector species. In this region, *An. gambiae* (*s.l.*) is known to breed in clear, temporary water bodies exposed to direct sunlight, whereas *An. funestu*s prefers semi-permanent to permanent water bodies with some degree of shading [40]. To be effective, bacterial larvicide interventions require that the habitats that produce malaria vectors are targeted continuously and for indefinite basis. Due to the ephemeral nature of *Anopheles* larval habitats, identifying and targeting these numerous larval habitats have been considered important challenges of larviciding interventions [13]. However, the advancements in geographical information system (GIS) technology, satellite imagery and drone-based multispectral imagery, have made mapping and generation of high-resolution geo-referenced landscape images possible [41–43]. With these new technologies, larval habitats can be relatively easily identified, mapped and targeted for larviciding, thereby overcoming the constraints of the traditional laborious methods of identifying and mapping the habitats.

Various bacterial larvicide products were tested in the reviewed studies, including most of the products available in the market or developed for mosquito control [12, 17]. WDG and CG formulations were the most preferred and these were also used in the large-scale control programmes. While the CG were easily applied by hand and were suitable in larval habitats with dense vegetation, WDG had a lower application rate due to their high toxin content (measured in international toxic units per milligram, ITU/mg). These properties also had implications for transport and storage costs. The newly formulated bacterial larvicides based on granules, tablets and briquettes were designed to offer flexibility in the application in different larval habitat types which vary in their physical characteristics and larvae productivity [12]. The reviewed articles indicated that products based on *Bti* and/or *Bs* were fairly easy to apply by hand or with conventional sprayers depending on the formulation and habitat characteristics. Although the larvicidal activity of used products is known to vary with mosquito species, the reviewed studies indicated that they were generally effective in controlling *An. gambiae* (*s.l.*), *An. funestus* (*s.l.*) and culicine mosquitoes.

The findings of studies implemented in diverse ecological conditions across SSA indicated that at low application rates, bacterial larvicides based on *Bti* and/or *Bs* were effective in controlling malaria vectors. The reported effectiveness of these agents in mosquito control corroborates well with findings of other studies conducted elsewhere outside SSA [44–46]. It was found that *Bti* and/or *Bs* caused a reduction in larval density, vector density, vector biting and malaria transmission in most of the tested areas. However, due to their short duration of activity, repeated applications at weekly or bi-weekly intervals were required to sustain control. On the other hand, products based on sustained slow release formulations showed relatively high residual activity ranging from 3–6 months in selected larval habitats [47, 48]. Moreover, the reviewed studies showed that the efficacy and residual activity of *Bti* and/or *Bs* on malaria vectors varied considerably with the prevailing ecological settings of the study site, the test products, as well as the study design. The activity of *Bti* and *Bs* is known to be influenced by factors related to the target mosquito, larval habitat conditions and larvicide properties as reported elsewhere [17]. The inherent variation in their activity in different ecological settings needs to be taken into account when designing and scaling-up larvicide interventions.

Many typical larval habitats for malaria vector mosquitoes, particularly the all-important *An. gambiae* complex in SSA, are in nature transient and shifting [40]. Most of these habitats originate from a wide range of economic important human activities such as agriculture, construction and mining [49]. Although some of the habitats are relatively more permanent and may contain some water for the most of the year, their size and, more importantly, the location of water margin where most of mosquito breeding activities take place fluctuates from week to week depending on weather conditions (mainly rain and sun). In addition to natural forces of the weather, man-made habitats are constantly modified to serve the purpose of which were created for, during cultivation and resumption of construction or mining activities. These activities may end up creating more new habitats or eliminate some altogether. Thus, many active larval habitats for malaria mosquitoes are not always static, but sometimes dynamic and a moving target. For this reason, irrespective of the residuality of the product applied, treated sites must be visited on a regular basis to identify and treat new active larval habitats that may have arisen or to re-treat the existing ones which have been affected by human activities. For this case, residuality is less valuable than it would otherwise be because of the duration and nature of the habitats themselves. Although long-lasting, slow release formulations of larvicides are desirable, less persistent conventional products have wide application in tropical weather conditions and more appropriate for the transient *An. gambiae* complex larval habitats.

Although the reviewed studies demonstrated the effectiveness of the bacterial larvicides based on *Bti* and *Bs* in malaria vector control, the products were found to be less effective in riverine areas with extensive flooding in The Gambia [50], in rice fields and swamps in Zaire (now The Democratic Republic of the Congo) [51], in transient rain puddles in Burkina Faso [27] and in overgrown wetlands in Tanzania [23]. These findings support the view that manual application of bacterial larvicides *Bti* and/or *Bs* by ground teams is not a strategy for all larval habitat types [14]. However, as malaria prevalence continues to decline, high transmission areas are attaining low to focal transmission, creating more conducive conditions appropriate for larviciding intervention. If sustained, the decline in malaria parasite transmission intensity creates an important opportunity for adoption of larviciding as a supplementary measure, though the strategy may not be suitable as a stand-alone intervention in many transmission settings. Moreover, it was evident from the reviewed studies that effective control of mosquito larvae can be achieved with repeated treatment of breeding sites and that malaria vector control with bacterial larvicides demands much greater ecological information with regard to water quality and the nature of the mosquito breeding habitats. It was also evident that larvicide intervention was more cost-effective in urban than in rural areas. To be effective, larviciding intervention with *Bti* and *Bs* needs to be well adapted to the prevailing local malaria vectors and their ecology.

High implementation costs have been considered as the main factor that prevents broader use of larvicide interventions particularly in SSA [13, 52]. Despite variation in the cost of these interventions reported in different areas of SSA, the cost compared favorably with those for LLINs and IRS interventions [53–55]. Some of the reviewed studies indicated that the interventions based on *Bti* and/or *Bs* were readily accepted by the general community in the intervention areas [23, 28–31]. In addition to participation in the larvicide applications, some community members indicated willingness to pay for the intervention [28–31]. Moreover, the safety of the tested products was found to be high [23–26], targeting only mosquito larvae and with no deleterious effect to non-target organisms, as also reported in other studies conducted outside SSA [56–58]. However, one of the reviewed articles reported that *Bti* treatment caused mortalities in Psychodidae larvae when applied at the recommended rate [27]. Species of the Psychodidae are among the dipterans that have been shown to be affected by *Bti* treatment as summarized elsewhere [59].

Larviciding intervention, particularly for control of mosquito with diverse breeding habitats like the major malaria vector in SSA, is labor-intensive undertaking. To be effective, larviciding teams must cover a large number of *Anopheles* larval habitats, some of which appear and disappear frequently in space and time with high-cost implications. In SSA, financial resources (for equipment, supplies and personnel costs) required to manage larviciding programmes remain intermittent and unreliable [60, 61]. Thus, mobilizing reliable sources of funding is crucial for the success of larvicide interventions. It was also found out that larvicides application by ground teams in rural areas with extensive larval habitats was inappropriate [50]. In areas with large flood plains, extensive wetland and rice cultivation which are largely inaccessible on foot, aerial application of larvicides is more appropriate. Further analysis of the reviewed field studies has shown that a wide range of larvicide products including non-WHOPES-recommended products [14] was evaluated in SSA (Tables 6, 7). This variation in product types tested coupled with heterogeneity in test conditions, did not permit unambiguous analysis of the efficacy of the interventions by geographical settings and/or malaria vector species. While more countries in SSA are adopting (larval source management, LSM) for control of malaria, it is crucial that a package of products for larviciding is streamlined based on WHOPES recommendations to allow for appropriate scale-up of the intervention in the future.

Apart from financial constraints, other major obstacles to delivering larviciding interventions to local communities in SSA include scarcity of trained personnel in the field of vector ecology/biology and lack of organizational structures for governance and management of vertical, decentralized LSM programmes [39, 62]. While it is crucial that these rare specialties are developed, a partnership between academic/research institutions and communities have been applied effectively to fill this technical gap in the implementation of larviciding or other vector control programmes in SSA [60, 62]. With the renewed interest in LSM in SSA, there is an urgent need to develop operational teams and robust organization structures for governance of these programmes in the near future [39, 60, 63]. Beside the outlined shortfalls, SSA is better positioned with adequate human resources to manage labor-intensive larval source management programmes. The availability of effective and yet safe microbial larvicides is making larviciding interventions feasible with such community-based staff with a minimal level of training.

Based on the effectiveness of larvicide products reported in the reviewed studies (Tables 6, 7), the historical success of larvicide interventions for malaria vector control [8, 11] and expert opinions [13, 37], larviciding remains a feasible option that can be included in the IVM programmes to supplement other vector control tools. Following decades of neglect of this strategy in SSA, research is still needed to improve quality of evidence and build skills especially in areas of malaria vector ecology, designing, monitoring and evaluation of larval source management programmes.

## Conclusions

The findings from the reviewed studies indicated that, at low application rates, bacterial larvicide products based on *Bti* and *Bs* were effective against malaria vectors in SSA. Furthermore, the larvicide intervention was found to be feasible and safe to non-target organisms and its cost compared fairly well with those of other vector control measures practiced in SSA. However, interventions based on these agents require substantial knowledge of larval ecology due to the effect of environment and larval habitat characteristics on these agents. As malaria continues to decline in SSA, larviciding should gain more ground due to shrinking of transmission areas and creation of more appropriate conditions for the intervention, and in order to delay the evolution of insecticide resistance and behavioral adaptations by the vectors. Moreover, the advancement of technology for mapping landscapes could facilitate the identification and targeting the numerous larval habitats preferred by the African malaria vectors. To build sustainable anti-larval measures in SSA, there is a great need to build capacity in relevant specialties in vector control and develop organizational structures for governance and management of larval source management programmes.

## Data Availability

All relevant data supporting the conclusions of this article are included within the article.

## Abbreviations

*Bti*: *Bacillus thuringiensis* var. *israelensis*
*Bs*: *Bacillus sphaericus*
ITU: international toxic units
WDG: water-dispersible granules
CG: corn granules
G: granules
FC: flowable concentrate
WP: wettable powder
SRG: sustained-release granular
WBS: water-based suspension
PP: primary powder
TP: technical powder
LC: liquid concentrate
AS: aqueous suspension
LLINs: long-lasting insecticide-treated nets
IRS: indoor residual spraying
SSA: sub-Saharan Africa
IVM: integrated vector management
GIS: geographical information system
EIR: entomological inoculation rate
PPPY: person protected per year
WHOPES: World Health Organization Pesticides Evaluation Scheme
LSM: larval source management
